# Rice Bran Extract Suppresses High-Fat Diet-Induced Hyperlipidemia and Hepatosteatosis through Targeting AMPK and STAT3 Signaling

**DOI:** 10.3390/nu15163630

**Published:** 2023-08-18

**Authors:** Joe Eun Son, Jay-Young Jo, San Kim, Min Ju Park, Yerin Lee, Seong Shil Park, Shin Young Park, Su Myung Jung, Sung Keun Jung, Ji Yeon Kim, Sanguine Byun

**Affiliations:** 1 Program in Developmental & Stem Cell Biology, The Hospital for Sick Children, Toronto, ON M5G 0A4, Canada; joeeun.son@sickkids.ca; 2 Department of Biotechnology, Yonsei University, Seoul 03722, Republic of Korea; jayyjo@yonsei.ac.kr (J.-Y.J.); syparksy@yonsei.ac.kr (S.Y.P.); 3 School of Food Science and Biotechnology, Kyungpook National University, Daegu 41566, Republic of Korea; 4 Research Institute of Tailored Food Technology, Kyungpook National University, Daegu 41566, Republic of Korea; 5 Department of Food Science and Technology, Seoul National University of Science and Technology, Seoul 01811, Republic of Korea; 6 Department of Biological Sciences, Sungkyunkwan University (SKKU), Suwon 16419, Republic of Korea

**Keywords:** rice bran extract, extraction optimization, lipid metabolism, hepatosteatosis, hyperlipidemia, AMPK, STAT3

## Abstract

Rice bran, a by-product of rice milling, is abundant in bioactive molecules and is highly recognized for its health-promoting properties, particularly in improving metabolic conditions. Building on this knowledge, we aimed to optimize the extraction conditions to maximize the functional efficacy of rice bran extract (RBE) and further validate its impact on lipid metabolism. We found that the optimized RBE (ORBE) significantly suppressed high-fat diet-induced weight gain, hyperlipidemia, and hepatosteatosis in mouse models. ORBE treatment not only suppressed lipid uptake in vivo, but also reduced lipid accumulation in HepG2 cells. Importantly, we discovered that ORBE administration resulted in activation of AMPK and inhibition of STAT3, which are both crucial players in lipid metabolism in the liver. Collectively, ORBE potentially offers promise as a dietary intervention strategy against hyperlipidemia and hepatosteatosis. This study underlines the value of optimized extraction conditions in enhancing the functional efficacy of rice bran.

## 1. Introduction

Rice (*Oryza sativa*) is a major global cereal crop. About half of the world’s population consumes rice as a dietary staple. Rice bran, a major by-product of rice milling, is produced in large quantities, exceeding 60 million tons, worldwide [1]. Often underutilized, used as livestock fodder, or even considered agricultural waste, the comprehensive utilization of rice bran represents a potential opportunity to mitigate this waste [2].

The consumption of rice bran and its derivatives has been shown to have various health benefits in numerous studies, including antioxidant, anti-inflammatory, and neuroprotective effects, as well as metabolic improvements [3,4,5]. Its beneficial impact on metabolic health, in particular, has been substantiated in multiple clinical studies. As rice bran is rich in bioactive polyphenols—notably γ-oryzanol, tocopherols, and tocotrienols—it is increasingly acknowledged as a valuable resource for developing nutraceutical agents with a variety of health benefits [4,6,7,8]. Given these advantages, enhancing the beneficial effects of rice bran through optimized extraction and processing techniques, aimed at maximizing its bioactive components, could augment its physiological impact. Such improvements, along with the validation of its functional efficacy, could greatly expand the application and use of rice bran.

Lipid metabolism plays a crucial role in maintaining overall health and energy balance in the body [9]. Dietary lipids, once ingested, are converted into absorbable fatty acid molecules by lipases and are subsequently absorbed in the gastrointestinal tract [10,11]. However, the excessive intake of fat—often due to westernized diets high in fat content—can lead to an overabundance of lipids in the body, resulting in obesity and related metabolic abnormalities, such as hyperlipidemia and hepatosteatosis [12,13]. Hyperlipidemia is defined by elevated lipid levels in the bloodstream, increasing the risk of other metabolic disorders [14,15]. Hepatosteatosis, also known as fatty liver disease, is characterized by excessive fat accumulation in the liver. If unchecked, this condition can evolve into serious liver diseases, including, fibrosis, cirrhosis, and even cancer [16,17,18]. Given that these pathological conditions often manifest without noticeable symptoms over long periods, preventive strategies, including dietary intervention with effective nutraceutical agents, become particularly meaningful [15,19].

In this study, through optimizing the extraction conditions, we developed a rice bran extract with enhanced functional efficacy and high γ-oryzanol content. We assessed the impact of the optimized rice bran extract (ORBE) on lipid metabolism in both rodent models and HepG2 liver cells. Our findings indicate that ORBE improves lipid metabolism and liver health by mitigating high-fat diet-induced hyperlipidemia and hepatosteatosis in vivo, reducing lipid accumulation in HepG2 cells, and modulating key lipid metabolism pathways. These results suggest that ORBE, developed through our optimized extraction process, holds promise as a potential dietary intervention to improve lipid metabolism and liver health.

## 2. Materials and Methods

### 2.1. Chemical and Reagents

Iso-propyl alcohol (HPLC grade), ethyl acetate (HPLC grade), and acetic acid (HPLC grade) were acquired from Sigma-Aldrich (St. Louis, MO, USA). Hexane was obtained from Thermo Scientific HyClone (Logan, UT, USA). Petroleum ether was procured from Samchun (Pyeongtaek, Gyeonggi-do, Republic of Korea). γ-oryzanol was purchased from Selleck chemical (Houston, TX, USA).

### 2.2. Preparation of Rice Bran Extract

The rice bran powder was acquired from a local rice mill situated in Haenam-gun, Jeollanam-do, Korea. Subsequently, the rice bran powder underwent extraction using a reflux extractor (MTOPS, Gyeonggi, Republic of Korea) employing distinct extraction factors. The extraction conditions for the different samples, denoted as RBE (rice bran extract) 1 to RBE 13, were as follows: RBE 1: 80 °C, 50% EtOH, 8 h; RBE 2: 60 °C, 70% EtOH, 8 h; RBE 3: 40 °C, 50% EtOH, 2 h; RBE 4: 60 °C, 30% EtOH, 2 h; RBE 5: 40 °C, 30% EtOH, 5 h; RBE 6: 40 °C, 70% EtOH, 5 h; RBE 7: 60 °C, 50% EtOH, 5 h; RBE 8: 60 °C, 70% EtOH, 2 h; RBE 9: 60 °C, 30% EtOH, 8 h; RBE 10: 40 °C, 50% EtOH, 8 h; RBE 11: 80 °C, 50% EtOH, 2 h; RBE 12: 80 °C, 30% EtOH, 5 h; RBE 13: 80 °C, 70% EtOH, 5 h. The selection of these extraction conditions was determined using a Box–Behnken design, and the corresponding values for the extraction factors can be found in Table 1. Following the extraction, the rice bran extracts underwent centrifugation for 5 min at a speed of 2000× *g* and were subsequently filtered using filter paper. The resulting filtrate was concentrated using rotary evaporation equipment (BUCHI, Gyeonggi, Republic of Korea) and subsequently subjected to freeze-drying. All lyophilized extracts were stored at −20 °C until further analyses.

### 2.3. HPLC Analysis of γ-Oryzanol in Rice Bran Extract

The concentration of γ-oryzanol in rice bran extracts was determined using an HPLC 1200 series instrument (Agilent Technologies, Santa Clara, CA, USA). To extract γ-oryzanol, 1 g of rice bran extract was pretreated in a Soxhlet apparatus with 250 mL of petroleum ether at 80 °C for 8 h. Following the Soxhlet extraction, the solvent was removed from the extract using rotary evaporation. Hexane was added to the Soxhlet extracts and subjected to sonication. The resulting extract was then filtered using a syringe filter for HPLC analysis. Prior to analysis, the sample was diluted with hexane at a ratio of 1:50. Detection was performed at a wavelength of 325 nm. HPLC columns (5 μm) from GL Science Inertsil SIL 100 A (Gl Sciences Inc., Torrance, CA, USA) were employed, maintained at a temperature of 25 °C. The mobile phase consisted of a mixture of hexane, iso-propyl alcohol, ethyl acetate, and acetic acid (97.6:0.8:0.8:0.8 *v*/*v*/*v*/*v*), flowing at a rate of 1.0 mL/min. A 10 μL injection volume was used. To establish a standard curve, γ-oryzanol standards ranging from 100 ppm to 1 ppm were injected. The γ-oryzanol content in the rice bran extracts was calculated using the following formula:

$$\gamma -\mathrm{oryzanol}\;\mathrm{content}\;(\mathrm{mg}/g)=\frac{A\;\left(\frac{\mathrm{mg}}{L}\right)\times V\;\left(L\right)\times DF}{C\;\left(g\right)}$$

*A* = concentration of test solution;

*V* = volume of filtered Soxhlet extract;

*DF* = dilution factor;

*C* = amount of rice bran prepared for Soxhlet extraction.

### 2.4. In Vitro Lipase Assay

Lipase activity was assessed by measuring the release of oleic acid (OA) from triolein. To prepare the reaction mixture, 80 mg of triolein, 10 mg of phosphatidylcholine, and 5 mg of taurocholic acid were combined with 9 mL of 0.1 M N-Tris (hydroxymethyl)methyl-2-aminoethanesulfonic acid (TES) buffer (pH 7.0) containing 0.1 M NaCl. The mixture was sonicated for 5 min. For the lipase activity measurement, the reaction mixture was assembled by adding 50 μL of enzyme solution (10 U/mL), 100 μL of sample extract (25 mg/mL), and 250 μL of substrate solution. The reaction mixture was then incubated at 37 °C for 30 min. Subsequently, 3 mL of a chloroform/n-hexane solution (1:1) containing 2% (*v*/*v*) methanol was added to the reaction mixture. The blend was stirred for 10 min and centrifuged at 2000× *g* to separate the supernatant. Next, 1 mL of a copper reagent was added to the supernatant, followed by shaking for 10 min and another centrifugation step (2000× *g*) for 10 min. The organic phase, which contained copper salts, was collected (0.5 mL), and 0.5 mL of a 0.1% (*w*/*v*) bathocuproine-chloroform solution containing methoxyphenol (10 mg) was mixed with it. The mixture was allowed to react for 10 min, and the absorbance was then measured at 480 nm using a microplate reader (BioTek Instruments, Inc., Winooski, VT, USA).

### 2.5. Cell Culture and Treatment

HepG2 hepatoma cells were obtained from the American Type Culture Collection. The HepG2 cells were cultured in Dulbecco’s Modified Eagle’s Medium (DMEM, Gibco, Billings, MT, USA) supplemented with 10% fetal bovine serum (FBS) at 37 °C with 5% CO_2_ and were allowed to reach 70–80% confluency. The cells were then treated with 0.6 mM oleic acid (#01257; Sigma, St. Louis, MO, USA) with or without RBE, then harvested for downstream molecular and imaging analysis.

### 2.6. Cell Viability Assay

A cell viability assay was conducted using the 3-(4,5-dimethylthiazol-2-yl)-2,5-diphenyltetrazolium bromide (MTT) assay. Briefly, HepG2 cells (1 × 10⁵ cells/mL) were seeded in a 96-well plate and cultured for 24 h. The cells were then treated with various samples at a concentration of 50 μg/mL or the indicated concentrations of RBE 2. After a 24 h incubation period, MTT solution was added to each well, followed by a 2 h incubation. Subsequently, the formazan crystals were dissolved using dimethyl sulfoxide, and the absorbance was measured at 560 nm using a microplate reader.

### 2.7. Cellular Triglyceride (TG) Content

HepG2 cells (5 × 10⁵ cells/well) were seeded in a 6-well plate and cultured for 24 h. The cells were then treated with serum-free media containing an OA solution (0.6 mM) and samples (50 μg/mL) for 24 h. After the treatment, the cells were washed with ice-cold PBS. Subsequently, the cells were lysed using a mixture of chloroform and methanol (2:1, *v*/*v*) (3 mL), and the lysates were vortexed and centrifuged (3000 rpm, 10 min, 4 °C). Following centrifugation, the organic phase was collected, and the solvent was evaporated under nitrogen gas until dry. The resulting pellet was dissolved in PBS containing 1% Triton X-100 to determine the cellular TG content using a TG kit (Asan pharmacology, Seoul, Republic of Korea).

### 2.8. Animals

Six-week-old male C57BL/6 mice were purchased from Orient Bio (Seongnam, Republic of Korea). The mice were acclimated for one week under a 12 h light/12 h dark cycle before the start of the experiments. After acclimation, the mice were provided with ad libitum access to water and to each diet including a normal chow diet, high-fat diet (HFD, D12451), or HFD supplemented with 1% rice bran extract. Five-week-old male Sprague–Dawley rats (SD rats) were obtained from Hana Biotech (Pyeongtaek, Republic of Korea) and underwent one week of acclimation at the Southeast Medi-Chem Institute (Busan, Republic of Korea) before being used for the experiments. The rats were kept under a 12 h light/12 h dark cycle and were provided with standard rodent diet and sterile water ad libitum.

### 2.9. High-Fat Diet Mouse Model

The 45% high-fat diet (D12451) was obtained from Research Diets, Inc., and 1% rice bran extract-supplemented high-fat diet was formulated using the aforementioned product. The experimental mice were randomly divided into three groups, with each group consisting of seven mice. Group I was fed with normal chow diet, group II was fed with the 45% high-fat diet, and group III was fed with the 45% high-fat diet supplemented with 1% rice bran extract. After the acclimation period, the animals were provided with their respective diets, and their body weights and food intakes were measured on a weekly basis. At the end of the experiment, the mice were fasted for 6 h before blood glucose level detection. Subsequently, the mice were anesthetized with CO_2_ for sacrifice. Organs, including livers, blood, and adipose tissues, were extracted, weighed, snap-frozen in liquid nitrogen, and stored at −80 °C until further use. Parts of the livers were utilized for histological analyses. The experiment was conducted in accordance with the guidelines and regulations set by the Institutional Animal Care and Use Committee of Yonsei University (IACUC-A-202109-1339-01, 1 November 2021).

### 2.10. In Vivo Lipid Uptake Assay, Oral Lipid Tolerance Test

Following an acclimation period, the rats were randomly assigned and distributed into three distinct groups (n = 7 per group): Group I received the vehicle (PBS), group II received corn oil (5 mL/kg), and group III received a combination of corn oil (5 mL/kg) and rice bran extract (0.5 g/kg). Prior to the experimental procedures, the animals underwent an overnight fasting period, after which they were orally administered with either rice bran extract or PBS, based on their respective group assignments. Subsequently, at the designated time point, corn oil or PBS was administered as per the experimental protocol, and blood samples were collected from the tail veins for subsequent analysis of plasma triglyceride levels. The experiment was organized within the guidelines and regulations of the Institutional Animal Care and Use Committee of the Southeast Medi-Chem Institute (SEMI-21-020, 9 December 2021).

### 2.11. Plasma Analysis

Plasma samples were subjected to analysis using an automatic biochemical analyzer (#7020; Hitachi, Tokyo, Japan) to quantify triglyceride (TG), total cholesterol (TC), low-density lipoprotein (LDL), high-density lipoprotein (HDL), aspartate transaminase (AST), and alanine aminotransferase (ALT) levels.

### 2.12. Gene Expression Analyses by Quantitative PCR

Total RNA was extracted from cells and tissue using the RNeasy Mini (Qiagen) or Purelink RNA Micro Kit (Thermofisher, Waltham, MA, USA), and complementary DNA was synthesized using M-MLV reverse transcriptase (Thermofisher) with oligo(dT). A gene expression assay was conducted using SYBR Green methods on Viia7 (Applied Biosystems, Waltham, MA, USA), and relative cycle threshold (CT) values were normalized by *ACTB* (β-actin). The following primers were used to detect expression: (*mCd36*: Fwd 5′-GATGGCCTTGCTTGGGATTGGA-3′, Rev 5′-GGCTTTACCAAAGATGTAGCCAGTG-3′; *mFabp4*: Fwd 5′-AAGTGGGAGTGGGCTTTGC-3′, Rev 5′-GGCTTTACCAAAGATGTAGCCAGTG-3′; *mPparg*: Fwd 5′-TGGGGATGTCTCACAATGCC-3′, Rev 5′-GATCTCCGCCAACAGCTTCT-3′; *mSrebp1*: Fwd 5′-GAACAGACACTGGCCGAGAT-3′, Rev 5′-TGAGCTGGAGCATGTCTTCG-3′; *hCD36*: Fwd 5′-AGTGATGATGAACAGCAGCAACA-3′, Rev 5′-CCTCAGCGTCCTGGGTTACAT-3′; *hFABP4*: Fwd 5′-AGGAATTTGACGAAGTCACTGCA-3′, Rev 5′-TGATTTTCCATCCCATTTCTGC-3′; *hPPARG*: Fwd 5′-ATGCTGGCCTCCTTGATGAA-3′, Rev 5′-TCACCAAAAGGCTTTCGCAG-3′; and *hSREBP1*: Fwd 5′-GCGGACAACCCATAATATCATTG-3′, Rev 5′-GCATCTTGGCGTCTGTCCC-3′).

### 2.13. Oil Red O Staining

Liver tissue samples were fixed in 4% paraformaldehyde, embedded in paraffin, and cut to 5 mm thick sections. Lipid droplets were visualized by staining with Oil Red O solution (0.5% in isopropanol, Sigma). Confluent HepG2 cells were treated with 0.6 mM oleic acid (#01257; Sigma) with or without RBE for 24 h. After 24 h, cells were fixed in 10% formalin and stained in freshly prepared Oil Red O solution (0.5% in isopropanol, Sigma). For lipid quantification, the dye was extracted from the stained cells by isopropanol with 4% NP-40, and absorbance was determined at 490 nm.

### 2.14. Immunoblotting

Liver tissues and HepG2 cells were lysed with RIPA buffer containing a protease (Bio-Rad Laboratories, Hercules, CA, USA) and phosphatase inhibitor cocktail (Sigma-Aldrich). Whole-cell lysates were obtained by sonication and centrifugated at 13,000 rpm for 10 min at 4 °C. The protein concentration was determined using a Pierce BCA Protein Assay Kit (Thermo Fisher Scientific). Protein was separated using Bolt™ 4–12%, 10-well or 12-well precast gels (Thermo Fisher Scientific) and transferred to a nitrocellulose (NC) membrane (PALL Corporation, Port Washington, NY, USA). The NC membrane was incubated with a specific primary antibody at 4 °C overnight. Antibodies specific for detecting p-ACC (#3661), ACC (#3676), p-AMPK (#2535), AMPK (#5831), b-actin (#3700), p-STAT3 (#9145), and STAT3 (#12640) were purchased from Cell Signaling Technology. Vinculin (sc-25336) was purchased from Santa Cruz Biotechnology, Santa Cruz, CA, USA. The next day, the membranes were incubated with HRP-conjugated secondary antibody and then bands were detected with Western Lightning Plus-ECL (PerkinElmer, Waltham, MA, USA). Protein bands were visualized using an automatic X-ray film processor (JPI Healthcare, Seoul, Republic of Korea). A densitometry measurement was performed using the Image Lab 6.1 software (Bio-Rad).

### 2.15. Statistical Analysis

Statistical analyses were performed using the PRISM 6.0 software (GraphPad, Boston, MA, USA). Data are expressed as the means ± standard error of the mean (SEM). The statistical significance of differences among groups was determined by Student’s t-test or one-way analysis of variance (ANOVA) with post hoc analysis. When ANOVA indicated statistical significance, Tukey’s honestly significant difference (HSD) test was used to determine the significantly different means. A probability value of *p* < 0.05 was used as the criterion for statistical significance (* *p* < 0.05, ** *p* < 0.01).

## 3. Results

### 3.1. Preparation of Different Rice Bran Extracts (RBE) Using Multiple Extraction Conditions, and Their Functional Screening on Lipid Metabolism

**Figure 1:**
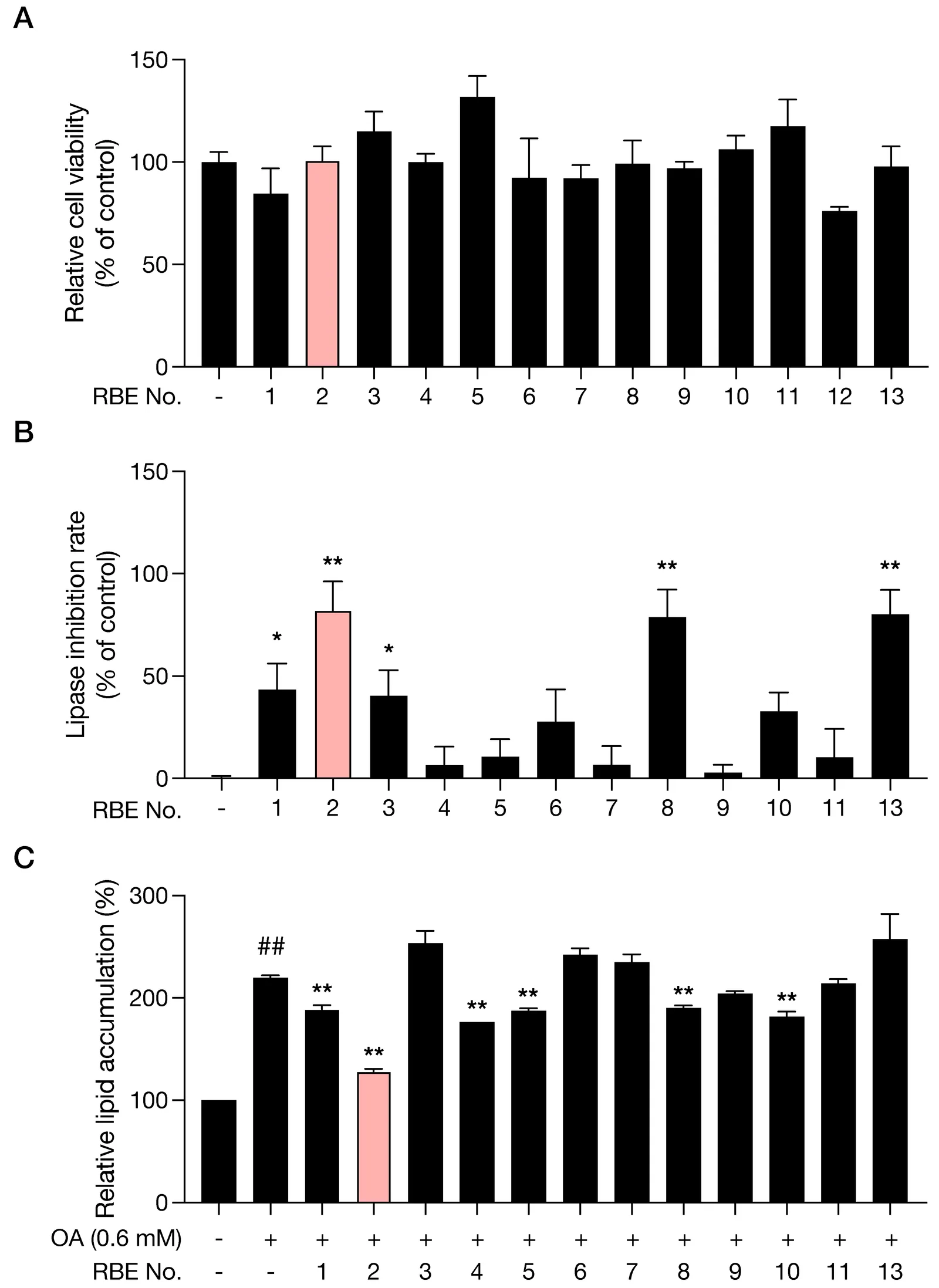
Functional screening of multiple rice bran extracts (RBEs) on lipid metabolism. (**A**) Cell viability of HepG2 cells was evaluated by MTT assay after 24 h of exposure to 50 μg/mL of each rice bran extract. (**B**) In vitro lipase inhibition assay was conducted with 2.5 mg/mL of each extract, and the inhibition rate was measured by comparing to the control. Data are represented as means ± SEM values. (* *p* < 0.05, ** *p* < 0.01 vs.—group). (**C**) Lipid accumulation was induced to HepG2 cells by treatment of 0.6 mM oleic acid with or without 50 μg/mL of each extract. After 24 h of treatment, cellular triglyceride content was quantified for each condition. RBE 2, which showed the most efficacy on both analyses without cytotoxicity, is highlighted in pink and was used as the optimized RBE (ORBE) in subsequent analyses. Data are represented as means ± SEM values. Statistical analyses were performed using one-way ANOVA with Tukey’s multiple comparison tests. (## *p* < 0.01 vs.—group; ** *p* < 0.01 vs. OA group).

Since extraction conditions can significantly influence the bioactivity of extracts [20], we applied multiple extraction conditions to produce thirteen distinct rice bran extracts (RBEs). To determine the most effective extraction conditions for controlling lipid metabolism, we evaluated the inhibitory effects of these extracts on lipase activity and lipid accumulation in the HepG2 liver cells (Figure 1A–C). Firstly, we assessed cell viability in the HepG2 cells following treatment with each RBE. RBE 12 was excluded from further experiments due to its cytotoxicity (Figure 1A). Next, we tested the lipase inhibition ability of the RBEs in vitro. RBEs 2, 8, and 13 demonstrated significant suppression of lipase activity (Figure 1B). Subsequently, we analyzed the impact of different RBEs on lipid accumulation induced by oleic acid in hepatocytes. RBE 2 exhibited the strongest inhibitory effect against oleic acid-induced lipid accumulation in HepG2 cells (Figure 1C). Additionally, we analyzed the concentration of γ-oryzanol in the extracts, a well-known active component of rice bran (Supplementary Table S1). Overall, among the RBEs, RBE 2 demonstrated the most potent efficacy in the conducted assays, leading to its selection for further analyses. As RBE 2 exhibited superior efficacy compared to other RBEs on both analyses, with the highest concentration of γ-oryzanol, we chose RBE 2 as the optimized RBE (ORBE) and further investigated its effect on lipid metabolism.

### 3.2. Oral Administration of ORBE Suppresses High-Fat Diet (HFD)-Induced Body Weight Gain and Related Hyperlipidemia in Mice

**Figure 2:**
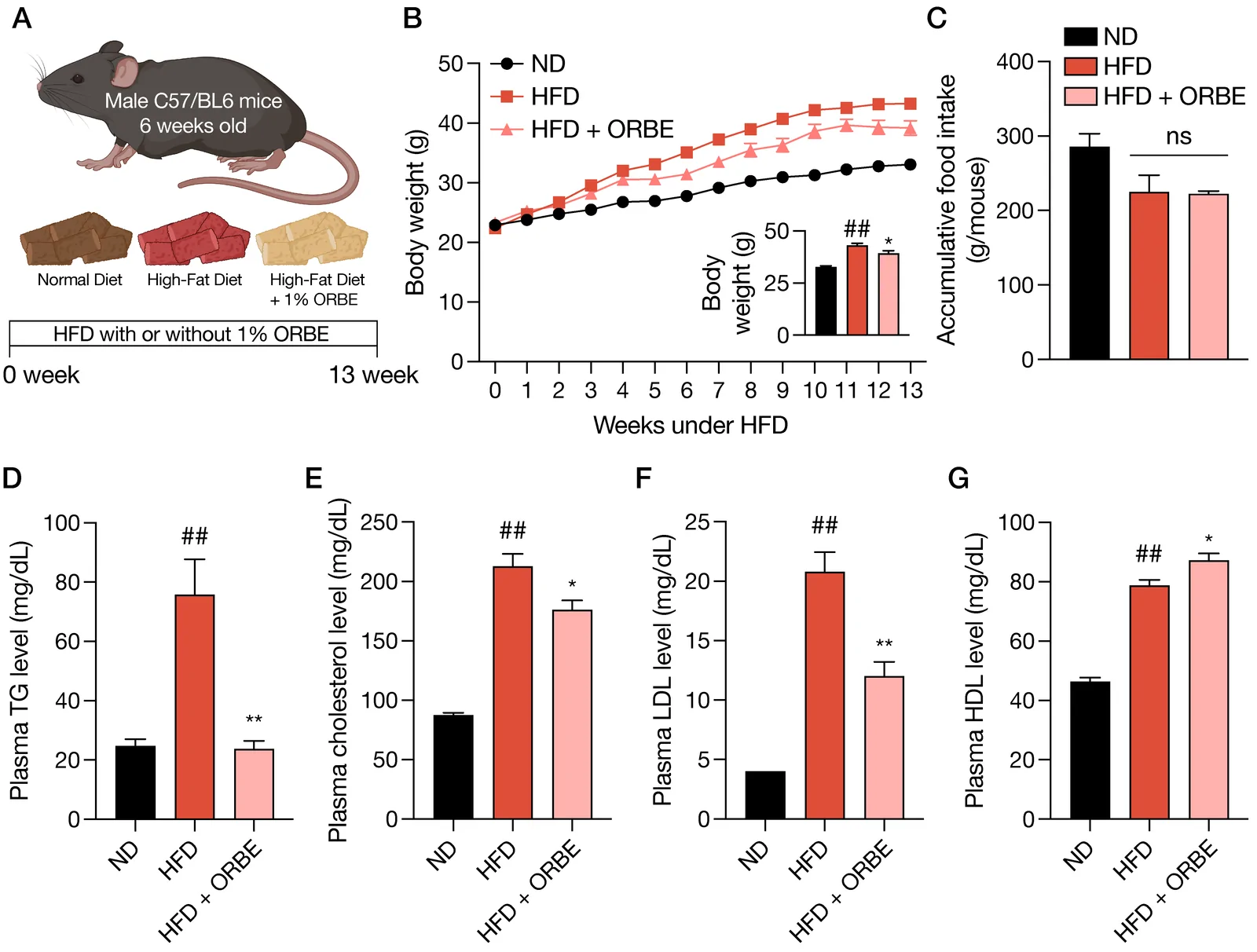
Effect of oral administration of ORBE on lipid metabolism in mice. (**A**) Experimental schematic depicting the high-fat diet feeding with or without 1% ORBE in mice. The schematic was created using illustrations from https://biorender.com, accessed on 1 June 2023. (**B**) Body weight or (**C**) food intake changes under HFD feeding with or without ORBE treatment. (**D**) Plasma triglyceride levels. (**E**) Plasma total cholesterol levels. (**F**) Plasma low-density lipoprotein levels. (**G**) Plasma high-density lipoprotein levels. Data are represented as means ± SEM values. Statistical analyses were performed using one-way ANOVA with Tukey’s multiple comparison tests. ns: not significant; ## *p* < 0.01 vs. ND group; * *p* < 0.05 or ** *p* < 0.01 vs. HFD group.

To investigate the in vivo effects of ORBE on metabolic improvement, mice were fed an HFD supplemented with 1% ORBE for 13 weeks (Figure 2A). Mice on the HFD displayed a dramatic increase in body weight compared to those on a normal chow diet (ND). However, the addition of ORBE significantly suppressed this HFD-induced weight gain (Figure 2B), with no observable difference in food intake compared to mice on the HFD alone (Figure 2C). Notably, mice administered with ORBE exhibited significantly lower levels of plasma triglyceride (TG), total cholesterol, and low-density lipoprotein (LDL), as well as higher levels of high-density lipoprotein (Figure 2D–G), suggesting that ORBE suppresses the dyslipidemia phenotype induced by the HFD. These results demonstrate that oral administration of ORBE leads to metabolic improvement, inhibiting HFD-induced weight gain and normalizing abnormal lipid levels in the blood.

### 3.3. Oral Administration of ORBE Protects Mice from HFD-Induced Lipid Accumulation in the Liver

**Figure 3:**
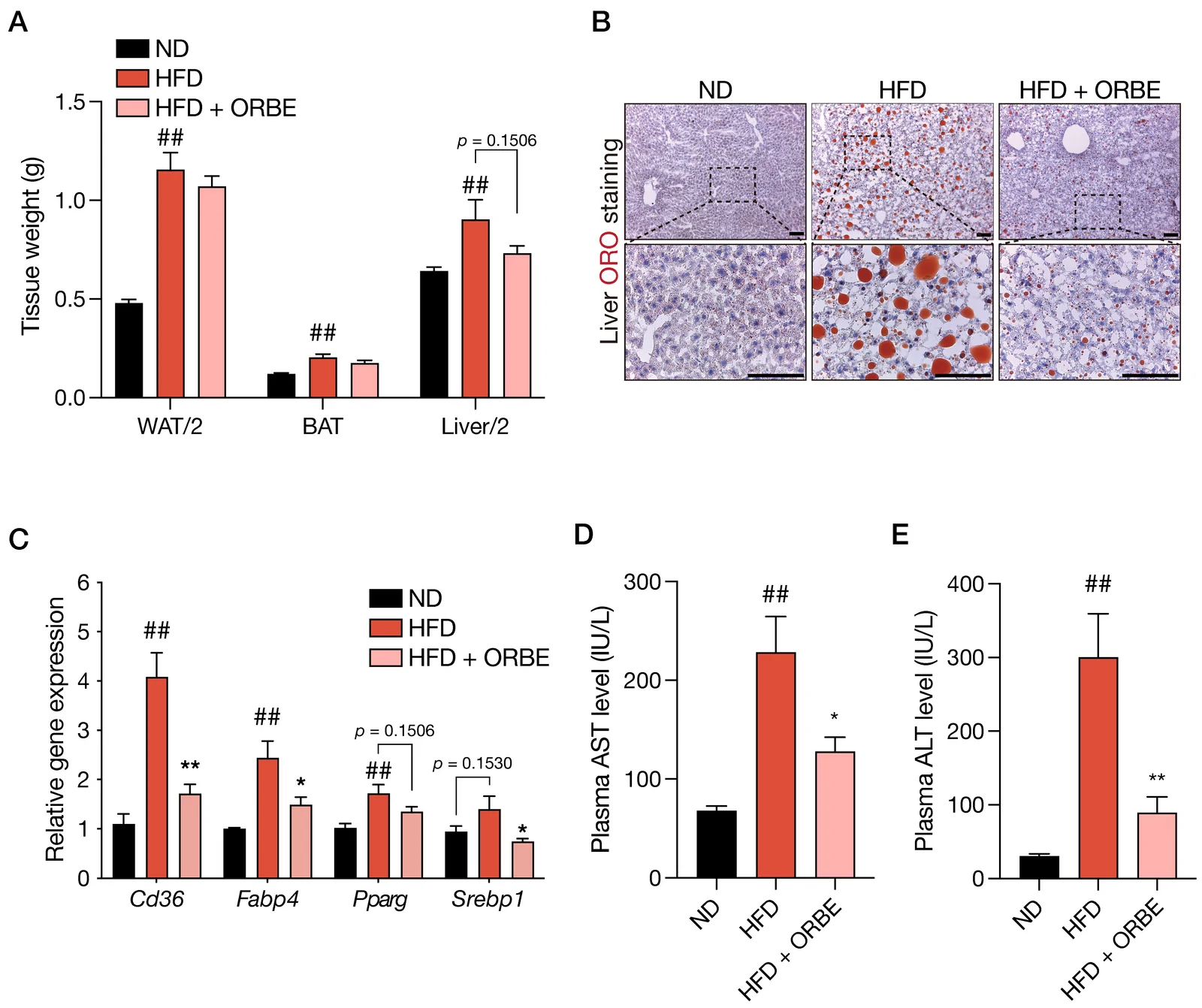
Oral administration of ORBE protects mice from HFD-induced hepatosteatosis. (**A**) Tissue weight of white adipose tissue (WAT) and brown adipose tissue from ND-fed mice, HFD-fed mice, and HFD + ORBE-fed mice. (**B**) Plasma aspartate aminotransferase (AST) activities, and (**C**) plasma alanine aminotransferase (ALT) activities from ND-fed mice, HFD-fed mice, and HFD + ORBE-fed mice. (**D**) Oil Red O (ORO) staining of liver sections. Scale bars, 100 μm. (**E**) RNA expression analysis of genes involved in lipid metabolism in liver. Data are represented as means ± SEM values. ## *p* < 0.01 vs. ND group; * *p* < 0.05 or ** *p* < 0.01 vs. HFD group.

In addition to its mitigating effect on hyperlipidemia, we found that ORBE treatment also prevented HFD-induced hepatic steatosis. Mice treated with ORBE exhibited lower liver weight, which was more pronounced than that seen in other major metabolic organs, such as white and brown adipose tissue, compared with HFD-fed mice (Figure 3A). These observations were further supported by the Oil Red O staining of liver sections, which revealed a noticeable reduction in lipid accumulation in the livers of ORBE-treated mice compared to those on an HFD (Figure 3B). Consistently, hepatic gene expression analysis exhibited that the HFD-induced upregulation of marker genes associated with lipid accumulation (*Cd36, Fabp4, PParg*, and *Srebp1*) was dramatically suppressed by ORBE treatment (Figure 3C). Additionally, ORBE treatment ameliorated the liver toxicity seen in HFD-fed mice, as illustrated by the significant reduction in plasma alanine aminotransferase (ALT) and aspartate aminotransferase (AST) levels compared to HFD-fed mice (Figure 3D,E). Our data suggest ORBE administration can protect HFD-induced hepatic steatosis and improve liver function, supporting the notion that ORBE treatment leads to metabolic improvement.

### 3.4. Oral Administration of ORBE Improves Lipid Handling In Vivo

**Figure 4:**
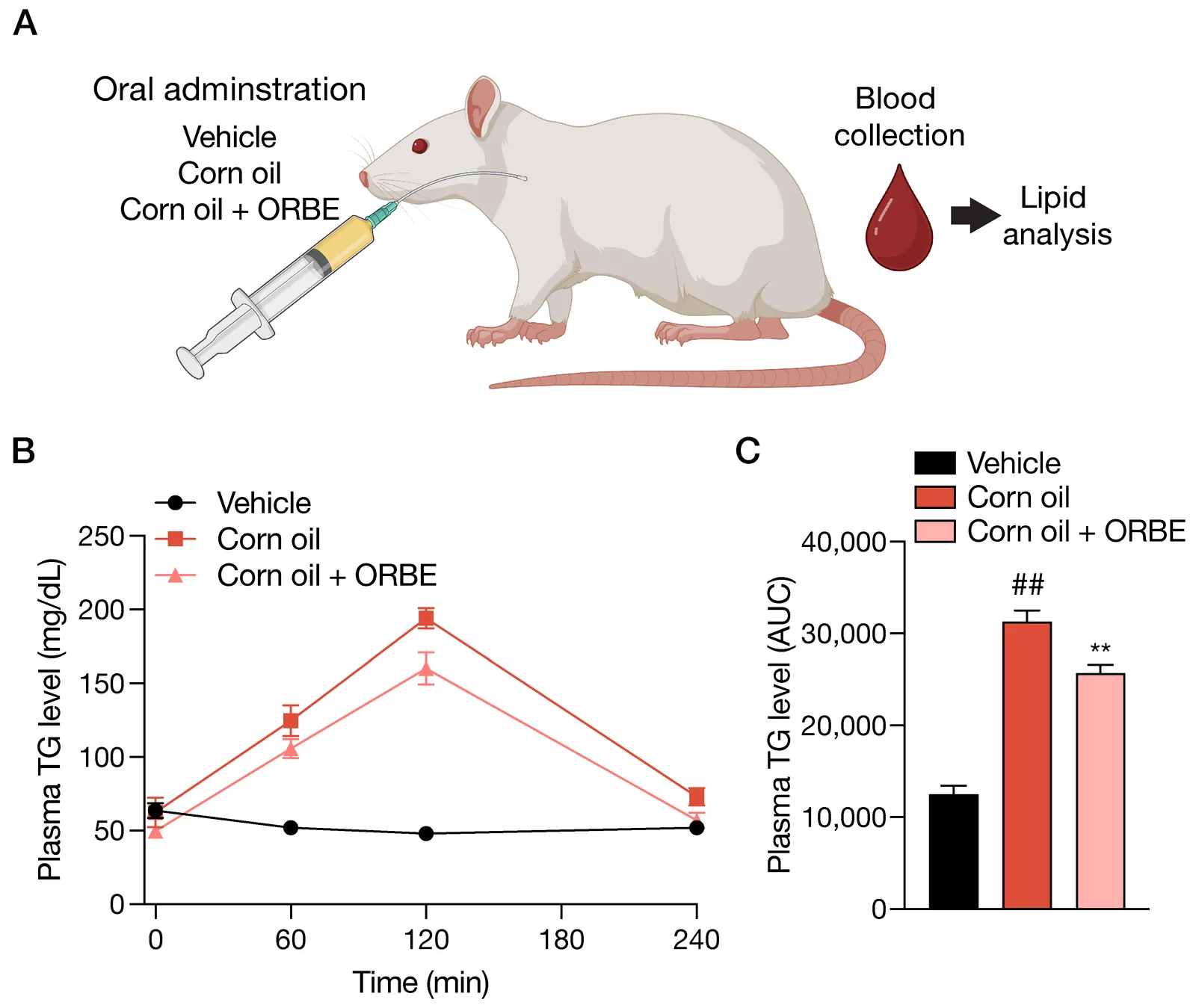
Oral administration of ORBE inhibits lipid uptake in vivo. (**A**) Experimental schematic of the acute lipid uptake assay in vivo. Plasma triglyceride (TG) levels were measured periodically after oral gavage of vehicle, corn oil (5 mL/kg), with/without ORBE (0.5 g/kg). The schematic was created using illustrations from https://biorender.com, accessed on 1 June 2023. (**B**) Plasma TG levels and (**C**) the bar graph shows the area under curve (AUC). Statistical analyses were performed using one-way ANOVA with Tukey’s multiple comparison tests. ## *p* < 0.001 vs. vehicle group. ** *p* < 0.01 vs. corn oil group.

Our observations that ORBE treatment substantially suppressed the hyperlipidemia phenotype in vivo led us to examine whether oral administration of ORBE could also influence lipid uptake. To assess this, we performed an oral lipid tolerance test (OLTT) using corn oil administered by gavage, both with and without concurrent ORBE administration (Figure 4A). Notably, OLTT demonstrated that increased plasma TG level by corn oil gavage was dramatically suppressed by ORBE administration with a smaller TG excursion (Figure 4B,C), suggesting that ORBE administration can suppress lipid uptake in vivo. Together with the in vitro lipase inhibition activity of ORBE (Figure 1B), these results collectively reveal that ORBE improves lipid handling capacity, leading to metabolic improvement.

### 3.5. Effect of ORBE on Lipid Accumulation and Related Gene Expression in HepG2 Liver Cells

**Figure 5:**
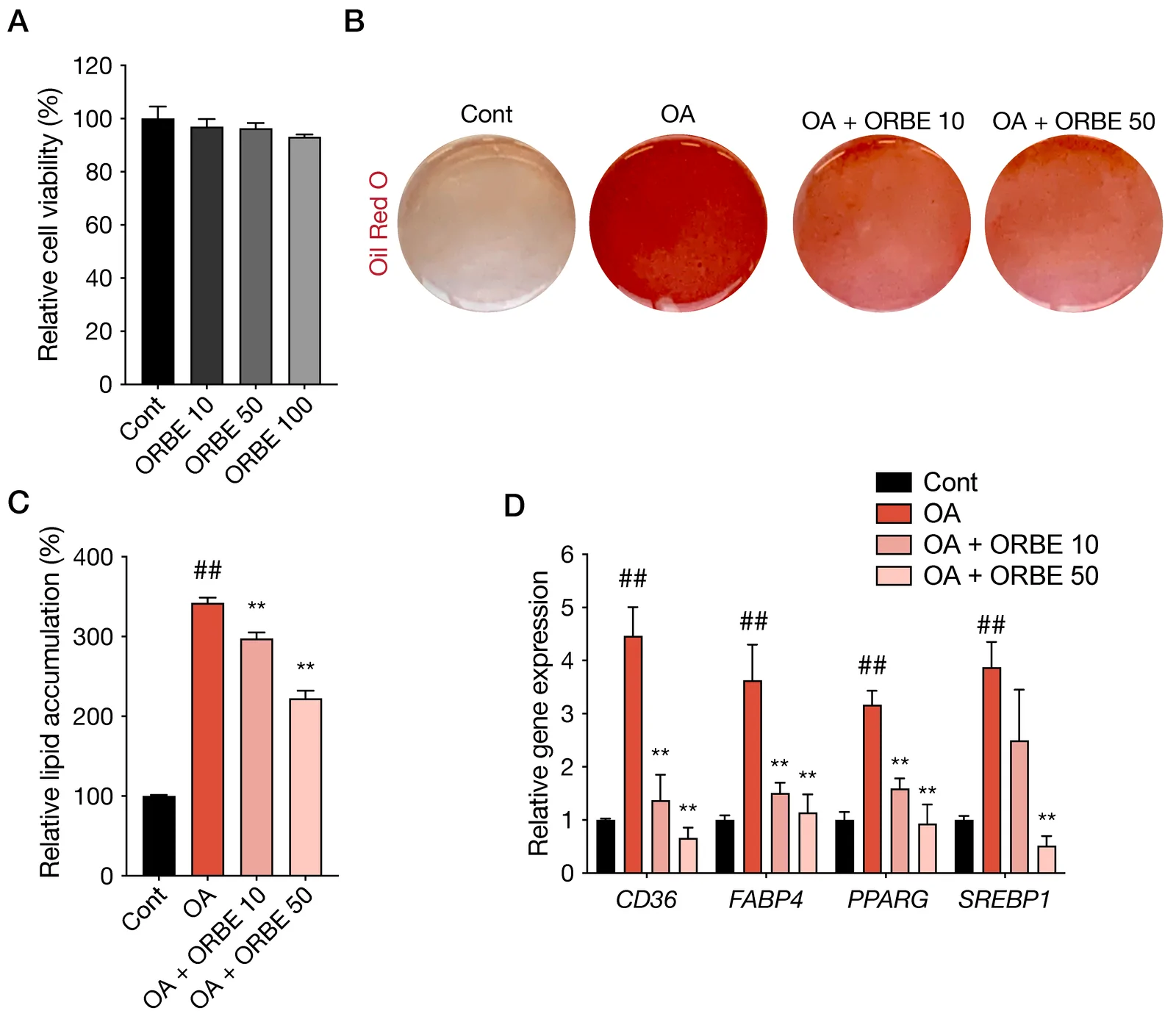
ORBE treatment ameliorates lipid accumulation in HepG2 liver cells. (**A**) Cell viability of HepG2 cells was evaluated by MTT assay after 24 h of exposure to ORBE. (**B**) Representative images of Oil Red O (ORO) staining in HepG2 cell culture dishes and (**C**) quantification of ORO staining after 24 h of exposure to oleic acid (OA) alone or OA in combination with ORBE. (**D**) Analysis of RNA expression levels of lipid metabolism-related genes in HepG2 cells after 12 h of exposure to either OA alone or OA combined with ORBE. Data are represented as means ± SEM values. Statistical analyses were performed using one-way ANOVA with Tukey’s multiple comparison tests. ns: not significant; ## *p* < 0.01 vs. control group; ** *p* < 0.01 vs. OA group.

Considering the potent preventive effect of ORBE on HFD-induced hepatic steatosis, we sought to further examine its hepatocyte-specific anti-steatotic effect using a HepG2 liver cell model of lipid accumulation. Lipid accumulation was induced in these cells by treating oleic acid, with or without concurrent ORBE treatment, within a dosage range that did not affect cell viability (Figure 5A). Oil Red O staining and subsequent quantification revealed that ORBE treatment markedly reduced oleic acid-induced lipid accumulation in a dose-dependent manner (Figure 5B,C), corroborating the in vivo observations of decreased hepatic lipid accumulation. Additionally, mRNA expression analysis of hepatic lipid accumulation marker genes (*CD36*, *FABP4*, *PPARG*, and *SREBP1*) showed a significant increase following oleic acid treatment, which was dose-dependently reduced by ORBE treatment (Figure 5D). These findings further solidify the anti-steatotic effects of ORBE by demonstrating its inhibitory action on lipid accumulation and related gene expression in HepG2 liver cells.

### 3.6. ORBE Treatment Activates AMPK Pathway and Inhibits STAT3 Activation in Mouse Liver and HepG2 Liver Cells

**Figure 6:**
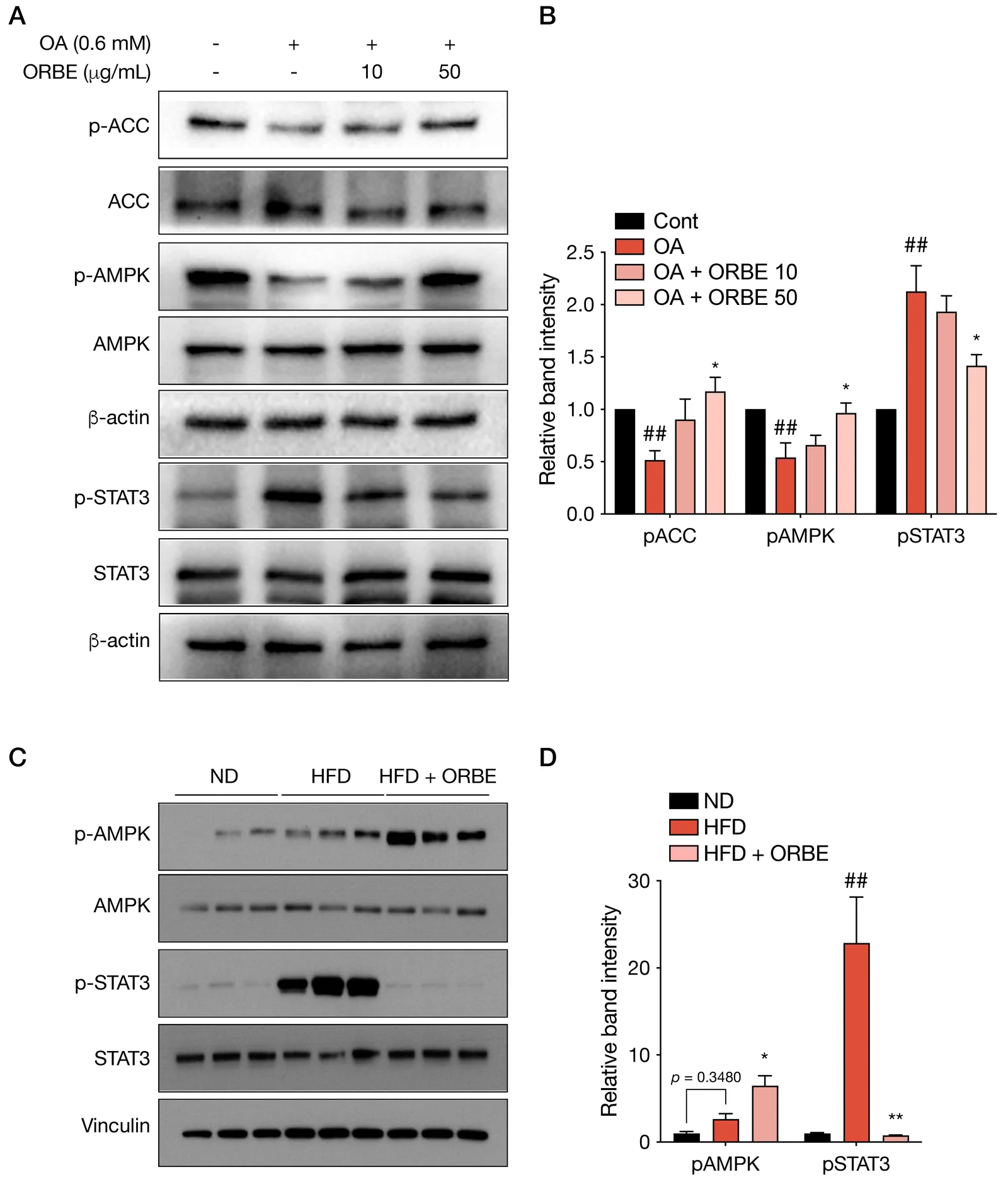
Effect of ORBE on signaling pathways in HepG2 liver cells and mouse liver tissue. (**A**) Representative band images and (**B**) quantification of the levels of phosphorylated and total ACC, AMPK, and STAT3 proteins in HepG2 cells after 6 h of exposure to either oleic acid (OA) alone or OA combined with ORBE, as determined by immunoblot analysis. Beta-actin was used as a loading control. (**C**) Representative band images and (**D**) quantification of the levels of phosphorylated and total AMPK and STAT3 proteins in mouse liver tissues as determined by immunoblot analysis. Vinculin was used as a loading control. Quantification of the levels of phosphorylated proteins was normalized with the level of total proteins. Data are represented as means ± SEM values. Statistical analyses were performed using one-way ANOVA with Tukey’s multiple comparison tests. (## *p* < 0.01 vs. control group; * *p* < 0.05 or ** *p* < 0.01 vs. OA group).

To decipher the underlying mechanisms by which ORBE inhibits hepatic lipid accumulation, we examined the effect of ORBE on cellular signaling pathways implicated in hepatic lipid metabolism. AMPK signaling is a known critical regulator of hepatic lipid metabolism, with several studies reporting its necessary activation for proper lipid metabolism in the liver [21,22,23]. Furthermore, stimulation by fatty acids results in a reduction in AMPK phosphorylation levels, indicating its deactivation in the liver under hypercaloric conditions [24,25,26]. Our immunoblot data revealed that oleic acid treatment downregulated the phosphorylation of ACC and AMPK, indicative of AMPK signaling deactivation (Figure 6A,B). Conversely, ORBE treatment significantly restored the phosphorylation of ACC and AMPK (Figure 6A,B), suggesting re-activation of AMPK signaling. In addition to the AMPK pathway, the STAT3 pathway is also critically involved in lipid metabolism and plays a pivotal role in the pathogenesis of liver diseases [27]. We found that oleic acid treatment induced STAT3 phosphorylation in HepG2 liver cells, whereas ORBE treatment dose-dependently suppressed OA-induced STAT3 phosphorylation (Figure 6A,B). Reinforcing the results observed in HepG2 cells, we also confirmed that in vivo ORBE administration significantly induced AMPK phosphorylation and dramatically inhibited STAT3 phosphorylation in mouse liver tissues compared to high-fat diet-fed mice (Figure 6C,D). These findings suggest that ORBE treatment upregulates the AMPK pathway and inhibits STAT3 activation in both mouse liver and HepG2 liver cells, leading to the amelioration of hepatic steatosis.

## 4. Discussion

In this study, we investigated the impact of optimized rice bran extract (ORBE) on lipid metabolism and hepatosteatosis. Our findings confirmed the potent effects of ORBE on ameliorating hyperlipidemia and reducing fatty liver disease in both in vitro and in vivo models. ORBE effectively mitigated high-fat diet-induced weight gain, suppressed hyperlipidemia, and lessened hepatosteatosis in mouse models. Moreover, we found that ORBE possesses strong lipase inhibitory activity, which in turn leads to the suppression of lipid absorption, contributing significantly to the management of hyperlipidemia. These beneficial effects were corroborated by in vitro studies, which showed reduced lipid accumulation in HepG2 cells following ORBE treatment. Future studies are warranted to investigate the detailed changes in the systemic profiles of lipids and related metabolites upon ORBE treatment. To address this question, a lipidomics/metabolomics analysis of liver tissue or plasma, with or without ORBE, would be the ideal experiment. Understanding the systemic changes in lipid metabolism upon ORBE treatment at the molecular level should yield deeper insights about its therapeutic potential, underlying mechanisms, and potential applications in dietary interventions or disease management.

While the metabolic improvement effects (e.g., anti-obesity, anti-hyperlipidemic or anti-hepatosteatosis) of RBEs are well documented [8,28,29,30,31,32,33,34,35,36,37,38], the optimal extraction conditions enhancing these biological activities have yet to be fully elucidated. As extraction conditions significantly affect bioactivity [20,39,40], identifying the optimal conditions and validating their functionality can enhance efficacy, reduce costs, and promote practical applications. In our study, we compared various extraction conditions, focusing on their impact on lipid metabolism. By modifying the extraction temperature, ethanol concentration, and extraction time, we observed considerable differences in the bioactivity of RBEs (Figure 2). For the solvent, we chose ethanol as a solvent due to its low toxicity, environmentally friendly nature, excellent polyphenol extraction efficiency, and suitability for large-scale processes [41]. Of all of the tested RBEs, RBE 2 showed the highest efficacy against lipase activity and lipid accumulation, containing the highest concentration of γ-oryzanol, known for its anti-obesity and anti-hyperlipidemic activities [29,42,43,44,45,46,47]. Interestingly, while RBEs 8 and 13 also exhibited comparable levels of γ-oryzanol, they did not yield a similar effect on lipid accumulation in HepG2 liver cells as RBE 2. This suggests that other bioactive compounds in RBE, possibly tocopherols or tocotrienols, might also contribute to the inhibition of lipid accumulation [48,49,50]. Further studies are warranted to detail the profile of bioactive compounds in ORBE and understand the roles of these compounds and their combined effects. Additionally, a deeper understanding of the absorption efficiency of ORBE and its primary active compounds is a critical avenue for future studies.

Our work has shed light on the potential molecular mechanisms through which ORBE exerts its beneficial effects on lipid metabolism in the liver. The AMPK pathway is a well-established regulator of hepatic lipid metabolism, with activation leading to a reduction in lipid accumulation [22,23]. We observed that ORBE effectively activated the AMPK signaling pathway, aligning with previous reports that suggest supplementation with rice bran phenolic extract ameliorates impaired lipid metabolism in HFD-fed mice through AMPK activation in the liver [28]. On the other hand, we also found that ORBE treatment resulted in the inhibition of STAT3 activation. STAT3, increasingly recognized for its role in cellular lipid uptake [27,51], has been shown to normalize lipid uptake when targeted, thus offering a potential target for addressing lipid-related metabolic disorders [52,53,54]. It is noteworthy that rice bran protein hydrolysates have previously been reported to inhibit the phosphorylation of STAT3 in HepG2 liver cells [55]. In line with these findings, our study indicates that ORBE could potentially regulate lipid accumulation and mitigate the progression of metabolic diseases by modulating the STAT3 pathway. These collective findings offer valuable insights into the molecular underpinnings of the metabolic improvements associated with ORBE treatment, providing a foundation for further exploration into its potential as a nutraceutical agent.

## 5. Conclusions

**Figure 7:**
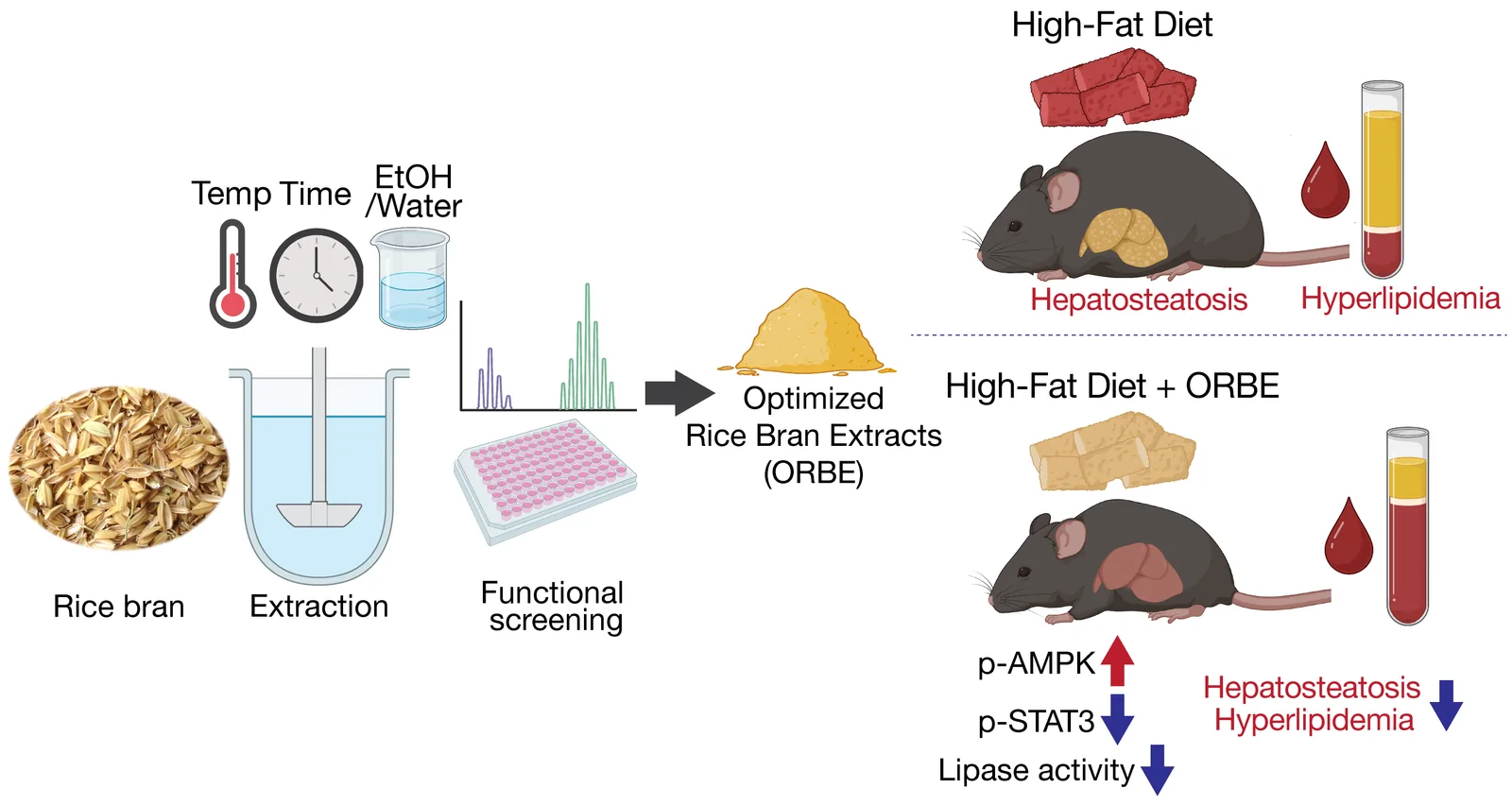
Schematic summary of the effects of ORBE on lipid metabolism and hepatosteatosis. The diagram represents the optimized extraction process of rice bran extract (RBE), leading to the optimized RBE (ORBE). Upon administration, ORBE shows potent suppression of lipase activity, leading to decreased lipid uptake and accumulation in vivo and in vitro. This suppression contributes to the reduction in HFD-induced weight gain, hyperlipidemia, and hepatosteatosis in mouse models. At the molecular level, ORBE activates the AMPK pathway and inhibits STAT3 activation in HepG2 cells and mouse liver. The schematic was created using illustrations from https://biorender.com, accessed on 1 June 2023.

In the current study, we have demonstrated that varying extraction conditions can significantly impact the bioactivity of rice bran extracts. Through optimizing the extraction conditions, we maximized the content of γ-oryzanol in RBE, a bioactive compound known for its metabolic benefits. Our findings showed that the optimized RBE (ORBE) significantly suppressed lipase activity, high-fat diet-induced weight gain, hyperlipidemia, and hepatosteatosis in mouse models, and also inhibited lipid accumulation in HepG2 liver cells. Furthermore, we revealed the underlying molecular mechanisms, with ORBE upregulating the AMPK pathway and inhibiting STAT3 activation, both crucial to lipid metabolism (Figure 7). These findings underline the value of optimized extraction conditions in enhancing the functional efficacy of rice bran extract and highlight its promise in combating lipid-related metabolic disorders.

## Figures and Tables

**Table 1:** The extraction factors and conditions applied for the preparation of rice bran extracts.

Extraction Factor	Level		
	−1	0	1
Temperature (°C)	40	60	80
Time (h)	2	5	8
Ethanol concentration (%)	30	50	70

## Data Availability

The data presented in this study are available on request from the corresponding author.

## Supplementary Materials

The following supporting information can be downloaded at: https://www.mdpi.com/article/10.3390/nu15163630/s1, Table S1: Comparison of Gamma-Oryzanol Concentration in Various Rice Bran Extracts.
