# Supplementary material for: Rice Bran Extract Suppresses High-Fat Diet-Induced Hyperlipidemia and Hepatosteatosis through Targeting AMPK and STAT3 Signaling
Source: Nutrients. 2023 Aug 18;15(16):3630. doi: 10.3390/nu15163630 (PMC10457887; doi:10.3390/nu15163630)
Supplement: Supplementary file 1 [file nutrients-15-03630-s001.zip › nutrients-2535898-supplementary.pdf]

nutrients MDPI

Supplementary Table S1: Comparison of Gamma-Oryzanol Concentration in Various Rice Bran Extracts.

Comparison of Gamma-Oryzanol Concentration in Various Rice Bran Extracts

RBE No.	gamma-oryzanol (mg/g)
1	0.4
2	12.27
3	0
4	0
5	0
6	9.33
7	0
8	10.82
9	0
10	0
11	0.75
12	0
13	11.09
